# Supplementary material for: A new AMPK isoform mediates glucose-restriction induced longevity non-cell autonomously by promoting membrane fluidity
Source: Nat Commun. 2023 Jan 18;14:288. doi: 10.1038/s41467-023-35952-z (PMC9849402; doi:10.1038/s41467-023-35952-z)
Supplement: Supplementary file 1 — Supplementary Information [file 41467_2023_35952_MOESM1_ESM.pdf]

SUPPLEMENTARY INFORMATION

A new AMPK isoform mediates glucose-restriction induced longevity non-cell autonomously by promoting membrane fluidity

Jin-Hyuck Jeong[1,2,7], Jun-Seok Han[1,7], Youngae Jung[3,7], Seung-Min Lee[1], So-Hyun Park[1,2], Mooncheol Park[1], Min-Gi Shin[1], Nami Kim[3], Mi Sun Kang[3], Seokho Kim[4], Kwang-Pyo Lee[1,2], Ki-Sun Kwon[1,5], Chun-A Kim[1], Yong Ryoul Yang[1], Geum-Sook Hwang[3,6] * and Eun-Soo Kwon[1] *

[1]Aging Research Center, Korea Research Institute of Bioscience and Biotechnology (KRIBB), Daejeon, 34141, Korea,

[2]Biomolecular Science, KRIBB School of Bioscience, Korea University of Science and Technology (UST), Daejeon, 34141, Korea,

[3]Integrated Metabolomics Research Group, Western Seoul Center, Korea Basic Science Institute, Seoul, 03759, Korea,

[4]Department of Medicinal Biotechnology, College of Health Sciences, Dong-A University, Busan, 49315, Korea,

[5]Functional Genomics, KRIBB School of Bioscience, Korea University of Science and Technology (UST), Daejeon, 34141, Korea,

[6]Department of Chemistry & Nano Science, Ewha Womans University, Seoul, 03760, Korea,

[7]These authors contributed equally to this work.

*Correspondence : gshwang@kbsi.re.kr, eunsoo.kwon@kribb.re.kr.

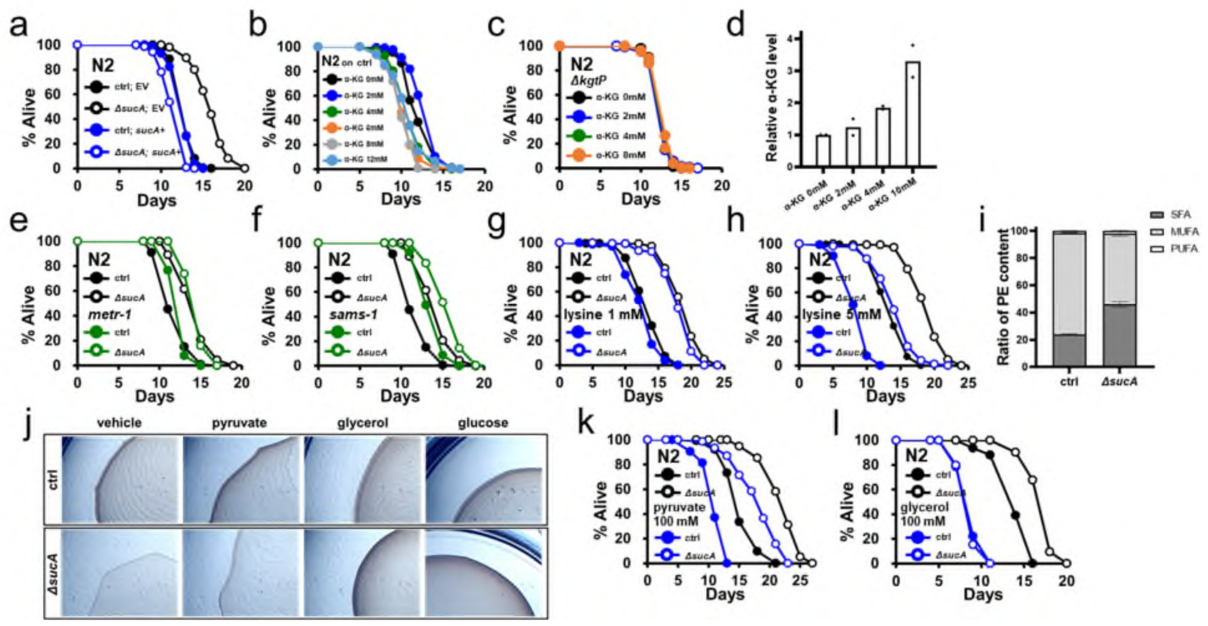

Supplementary Figure 1. GR-modulated lifespans under various conditions

a, Lifespan showing that wild-type *sucA*$^{+}$ complementation suppress the lifespan extension by *ΔsucA* mutation (*P* = 0.147, P value determined by two-tailed Student′s *t* test). Results from representative experiments are shown with additional repeats.

b, Lifespans showing that αKG supplementations (2, 4, 6, 8, 12mM) do not extend the *C. elegans* lifespan. Results from one of three independent experiments are shown.

c, Lifespan showing that αKG supplementations (2, 4, 8mM) do not extend the *C. elegans* lifespan fed *ΔkgtP E. coli* (*P* = 0.727, P value determined by two-tailed Student′s *t* test). Results from representative experiments are shown with additional repeats.

d, Assay of intracellular αKG measurement showed that increase in a dose-dependent manner. 2 biological repeats are used for analysis. bars indicate mean of relative αKG level.

e-f, Lifespan showing that GR diets extend the lifespan of *metr-1(ok521)* (e) and *sams-1(ok3033)*

mutants (f) ($P < 1.0* 10^{-10}$, P value determined by two-tailed Studentʹs *t* test). Results from representative experiments are shown with additional repeats.

g-h, Lifespan showing that lysine treatment (1mM, 5mM) does not extend lifespan of *C. elegans.* ($P < 1.0* 10^{-10}$, P value determined by two-tailed Studentʹs *t* test). Results from representative experiments are shown with additional repeats.

i, Relative saturation of acyl chains of phosphatidylethanolamine (PE) obtained from control, *ΔsucA E. coli* showed *ΔsucA E. coli* is increased saturated fatty acid (SFA) significantly. All results are presented as means ± SEM (independent biological repeats N=3).

j, DIC image showing differential growth of *ΔsucA E. coli* by pyruvate, glycerol and glucose. Representative photos from additional repeats are shown.

k, Lifespan showing that pyruvate treatment (100mM) does not affect lifespan extension by GR diets. ($P < 1.0* 10^{-10}$, P value determined by two-tailed Studentʹs *t* test). Results from representative experiments are shown with additional repeats.

l, Lifespan showing that glycerol treatment (100mM) completely suppressed GR-mediated lifespan extension. ($P= 0.3822$, P value determined by two-tailed Studentʹs *t* test). Results from representative experiments are shown with additional repeats.

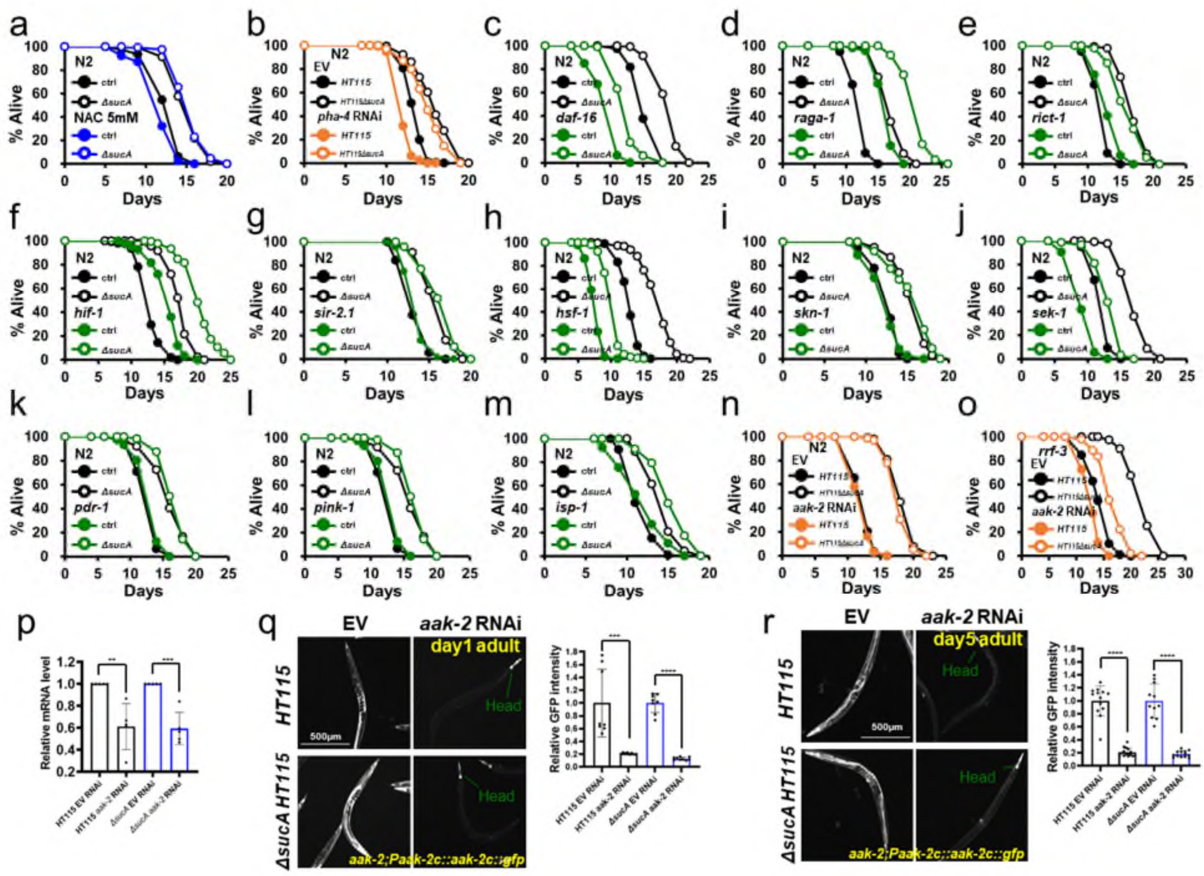

Supplementary Figure 2. GR-modulated lifespans of *C. elegans* mutants, transgenic animals, or RNAi animals

a, Lifespan showing that *N*-acetyl cysteine (NAC) does not affect lifespan extension by GR diets ($P < 1.0* 10^{-10}$, P value determined by two-tailed Student′s *t* test). Results from representative experiments are shown with additional repeats.

b-g, Lifespans showing that DR effectors are not required for GR-induced longevity ($P < 1.0* 10^{-10}$, P value determined by two-tailed Student′s *t* test). Results from one of at least two independent experiments are shown. The lifespan of *C. elegans* mutants presented are *pha-4*

RNAi (b), *daf-16(mgDf50)* (c), *raga-1(ok386)* (d), *rict-1(ft7)* (e), *hif-1(ia4)* (f), and *sir-2.1(ok434)* (g).

h-m, Lifespan showing that stress response and mitochondrial longevity pathway are not required for GR-induced longevity ($P < 0.001$, P value determined by two-tailed Student′s *t* test). Results from one of at least two independent experiments are shown. The lifespan of *C. elegans* mutants presented are *hsf-1(sy441)* (h), *skn-1(zu135)* (i), *sek-1(km4)* (j), *pdr-1(gk448)* (k), *pink-1(ok3538)* (l), and *isp-1(qm150)* (m).

n, Lifespan showing that *aak-2* RNAi does not reduce the lifespan extension by GR diets in wild type N2 ($P < 1.0 * 10^{-10}$, P value determined by two-tailed Student′s *t* test). Results from representative experiments are shown with additional repeats.

o, Lifespan showing that *aak-2* RNAi significantly attenuates the lifespan extension by GR diets in RNAi hypersensitive *rrf-3(pk1426)* animals. Results from representative experiments are shown with additional repeats.

p, Relative *aak-2* transcript levels showing that *aak-2* RNAi significantly reduces the expression of *aak-2* (mean ± s.d. of five biological replicates, $P = 0.0032$, $P = 0.0003$, respectively, P value determined by two-tailed Student’s *t* test). Total RNAs are obtained from day3 N2 animals grown on control and *aak-2* RNAi bacteria.

q-r, GFP images showing that *aak-2* RNAi significantly reduces the fluorescence intensity of *aak-2(ok524);aak-2c::gfp* transgenic animals. Results from representative experiments are shown with additional repeats. Right panels are quantifications. GFP intensity are quantified using ImageJ (Error bar indicate mean ± s.d., $n \geq 8$ for each condition, $P < 0.001$, EV RNAi vs.

aak-2 RNAi *P* = 0.0009 (q), P value determined by two-tailed Student′s *t* test).

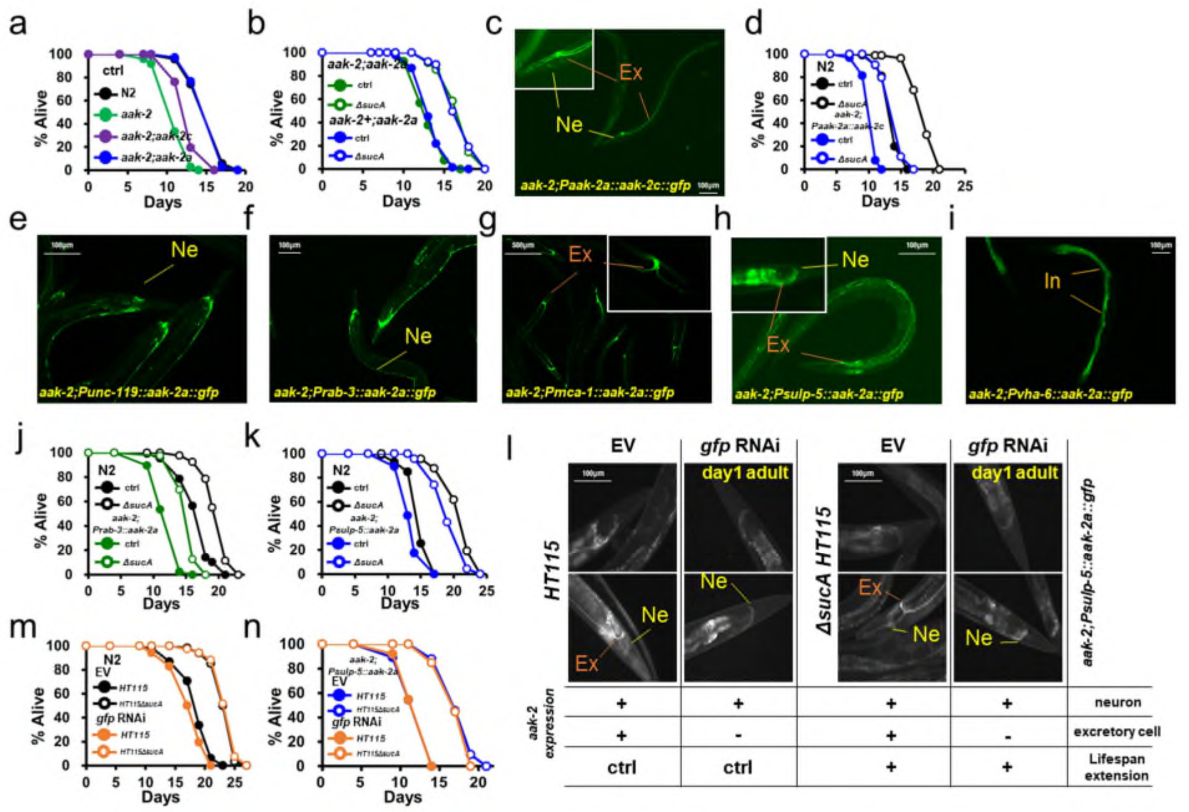

Supplementary Figure 3. Neuronal AMPK is required for GR-induced longevity

a, Lifespans showing the differential contributions to lifespan by AAK-2a and AAK-2c isoform. AAK-2a play the critical role in lifespan regulation. *aak-2(ok524);aak-2c* transgenic animals lived longer than *aak-2* mutant animals ($P < 1.0 * 10^{-10}$, P value determined by two-tailed Student's *t* test). Results from representative experiments are shown with additional repeats.

b, Lifespan showing that lifespan of *aak-2(ok524);aak-2a::gfp* animals are comparable with those of *aak-2*$^{+}$*;aak-2a::gfp* animals on either AL or GR diets. Results from representative experiments are shown with additional repeats.

c, GFP image showing the expression patterns of AAK-2c isoform driven by *aak-2a* promoter.

AAK-2c::GFP is expressed in head neurons (Ne) and excretory cells (Ex), similarly with AAK-2a. Representative images obtained from 5 biological independent repeats.

d, Lifespan showing that GR diets extended the lifespan of *aak-2(ok524);Paak-2a::aak-2c::gfp* animals ($P < 1.0* 10^{-10}$, P value determined by two-tailed Student′s *t* test). Results from representative experiments are shown with additional repeats.

e-i, GFP image showing the expression patterns of AAK-2a isoform. AAK-2a is expressed using tissue specific promoters, the pan-neuronal *Punc-119* (e), the pan-neuronal *Prab-3* (f), excretory *Pmca-1* (g), excretory *Psulp-5* (h), and intestinal *Pvha-6* (i) promoters in the *aak-2(ok524)* background. The *Psulp-5* driven *aak-2a::gfp* is in the excretory cells and neurons (h). Representative images obtained from at least 3 biological independent repeats.

j, Lifespan showing that GR diets extended the lifespan of *aak-2(ok524);Prab-3::aak-2a::gfp* ($P < 1.0* 10^{-10}$, P value determined by two-tailed Student′s *t* test). Results from representative experiments are shown with additional repeats.

k, Lifespan showing that GR diets extended the lifespan of *aak-2(ok524);Psulp-5::aak-2a::gfp* ($P < 1.0* 10^{-10}$, P value determined by two-tailed Student′s *t* test). Results from one of three independent experiments are shown.

l, GFP images showing that *gfp* RNAi specifically reduces the expression of *aak-2a* in excretory cells in day 1 adult *aak-2(ok524);Psulp-5::aak-2a::gfp* animals. Representative images obtained from 2 biological independent repeats.

m-n, Lifespans showing that GR diets extended the lifespan of wild type *C. elegans* (m) and *aak-*

2(ok524);Psulp-5::aak-2a::gfp (n) when knocked down using *gfp* RNAi ($P < 1.0*10^{-10}$, P value determined by two-tailed Student's *t* test). Results from representative experiments are shown with additional repeats.

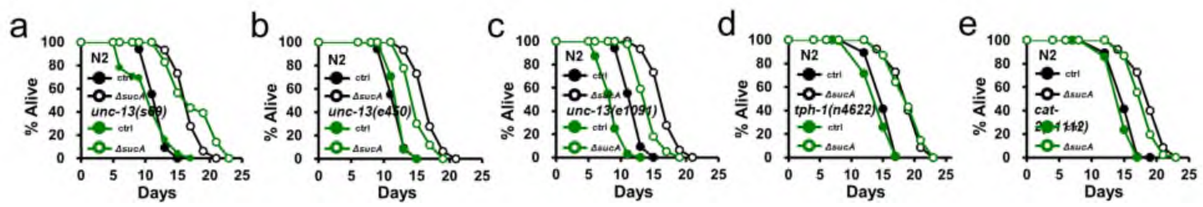

Supplementary Figure 4. Neurotransmitters are dispensable for GR-mediated longevity

a-c, Lifespans showing that multiple *unc-13* mutant alleles, including *unc-13(s69)* (a), *unc-13*(*e450)* (b), and *unc-13(e1091)* (c) are not implicated in GR-induced longevity (*P* < 1.0* 10^{-10}, P value determined by two-tailed Student′s *t* test). Results from representative experiments are shown with additional repeats.

d-e, Lifespans showing that GR diets prolong the longevity of mutants for serotonin, *tph-1(n4622)* (d), dopamine, *cat-2(e1112)* (e) (*P* < 1.0* 10^{-10}, P value determined by two-tailed Student′s *t* test). Results from representative experiments are shown with additional repeats.

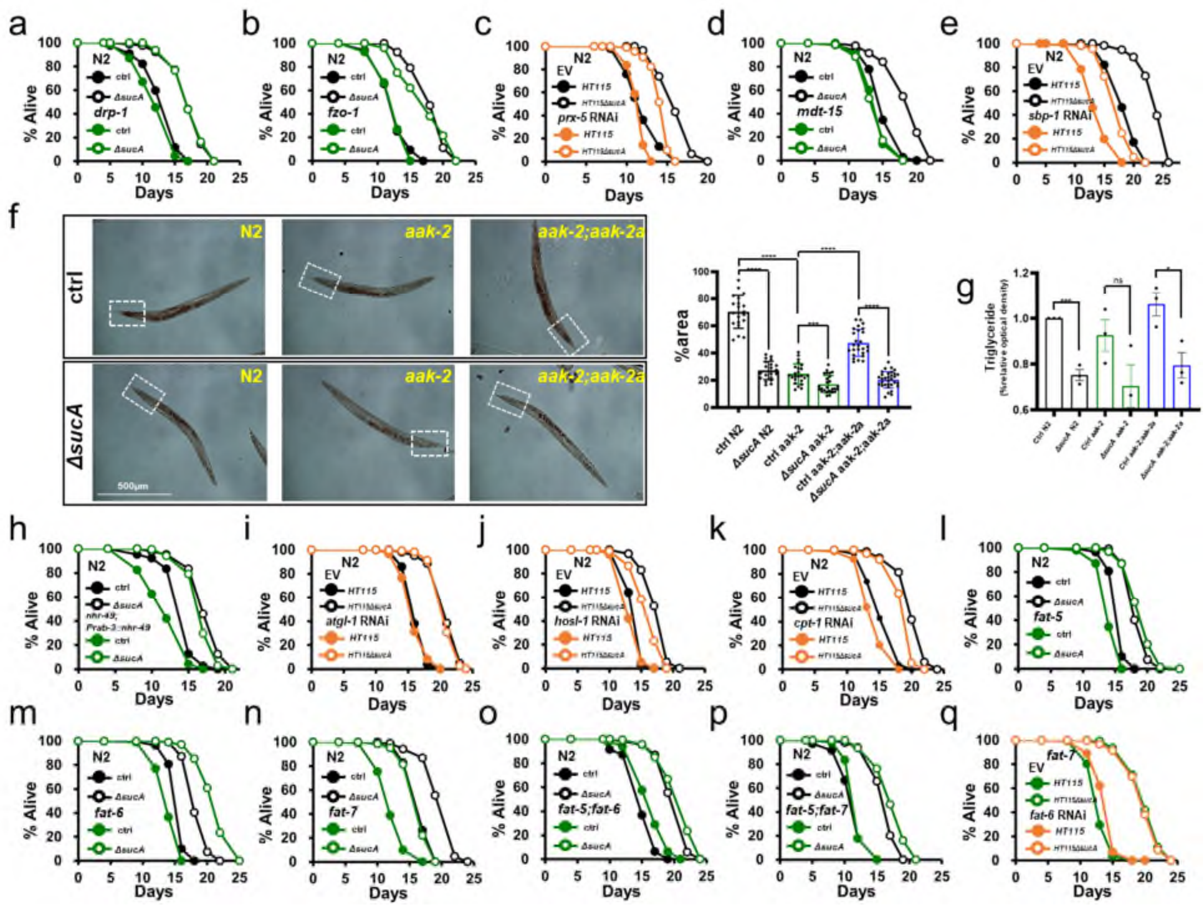

Supplementary Figure 5. GR-induced lifespans in mutants of organelle maintenance and fat metabolism

a-c, Lifespans showing that GR diets extend the lifespan of mitochondrial dynamics mutants, such as *drp-1(tm1108)* (a), *fzo-1(tm1133)* (b), and peroxisome depleted animals (*prx-5* RNAi) (c) ($P < 1.0* 10^{-10}$, P value determined by two-tailed Student's *t* test). Results from one of at least two independent experiments are shown.

d, Lifespan showing that *mdt-15(tm2182)* abolished GR-mediated longevity ($P = 0.7534$, P value determined by two-tailed Student's *t* test). Results from representative experiments are shown

with additional repeats.

e, Lifespan showing that *sbp-1* RNAi does not affect GR-mediated longevity

f, Fat store of day 1 animals feeding AL vs. GR diets. Fixed worms are stained with Oil Red O dye. Fat contents are quantified using the ImageJ software. Intensity of posterior parts of intestine are quantified as fat contents (dotted boxes) using ImageJ software (***$P < 0.001$, ****$P < 0.0001$, *aak-2* on AL vs. GR diets $P = 0.0003$, two-tailed unpaired Student's *t* test). Error bars indicate standard error of mean (SEM). Results from representative experiments are shown with additional repeats, $n \geq 20$ for each condition. Right panel is quantification.

g, Biochemical assays for triacylglycerol (TAG) levels in wild type N2, *aak-2(ok524),* and *aak-2(ok524);aak-2a::gfp* animals grown on AL vs. GR diets. 3 biological repeats are used for analysis, $n > 500$ for each condition. Error bars indicate standard error of mean (SEM).

h, Lifespans showing that *nhr-49(nr2041)* with pan-neuronal promoter *rab-3::NHR-49::GFP* recued GR-mediated longevity. Results from representative experiments are shown with additional repeats.

i-k, Lifespans showing that GR diets extended the lifespan of *C. elegans* when *atgl-1* (i), *hosl-1* (j), and *cpt-1* (k) was knocked down using RNAi ($P < 1.0 * 10^{-10}$, P value determined by two-tailed Student's *t* test). Results from representative experiments are shown with additional repeats.

l-q, Lifespans showing that GR diets extended the lifespan of *fat-5(tm420)* (l), *fat-6(tm331)* (m), *fat-7(wa36)* (n) single mutants ($P < 1.0 * 10^{-10}$, P value determined by two-tailed Student's *t* test).

The lifespan of each single mutant is tested once, as GR diets extend the lifespan of all double mutants combinations. GR diets extend the lifespan of *fat-5(tm420);fat-6(tm331)* (o), *fat-5(tm420);fat-7(wa36)* (p), *fat-7(wa36);fat-6*(RNAi) (q) animals ($P < 1.0 * 10^{-10}$, P value determined by two-tailed Student′s *t* test). Results from representative experiments are shown with additional repeats.

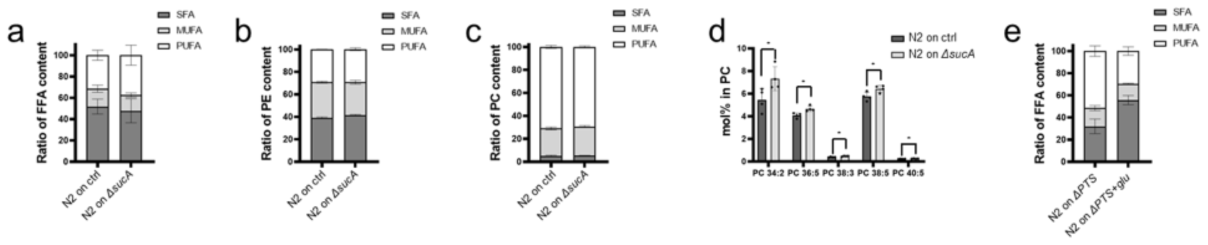

Supplementary Figure 6. GR diets modulate lipid composition

a, Relative saturation of free fatty acid (FFA) of *C. elegans* fed on AL vs. GR diets. All results are presented as means ± SEM (independent biological repeats N=4). Two-tailed Student's *t* test was used. See also Supplementary table 7.

b, Relative saturation of acyl chains of phosphatidylethanolamine (PE) obtained from *C. elegans* fed on AL vs. GR diets. All results are presented as means ± SEM (independent biological repeats N=4). Two-tailed Student's *t* test was used. See also Supplementary table 7.

c, Relative saturation of acyl chains of phosphatidylcholine lipids (PC) obtained from *C. elegans* fed on AL vs. GR diets. All results are presented as means ± SEM (independent biological repeats N=4). Two-tailed Student's *t* test was used. See also Supplementary table 7.

d, *C. elegans* phosphatidylcholine lipids (PC) composition changes fed on AL vs. GR diets. Each row indicates the fraction (mol%) of fatty acids in PC (**P* < 0.05, PC 32:1 P = 0.01178, PC 34:2 0.04259, PC 36:5 P = 0.02995, PC 38:5 P =0.02995, PC 38:6 P =0.00895, PC 38:9 P =0.0173, PC 40:5 P = 0.040, P value determined by two-tailed Student′s *t* test). All results are presented as means ± SEM (independent biological repeats N=4). Two-tailed unpaired Student's t test was used. See also Supplementary table 7.

e, Relative saturation of acyl chains of *C. elegans* free fatty acid (FFA) changes fed on *ΔPTS*^{Glc} vs. *ΔPTS*^{Glc} *E. coli* +2% glucose (SFA, PUFA, $P < 0.001$). All results are presented as means ± SEM (independent biological repeats N=3). Two-tailed Student's *t*-test was used. See also Supplementary table 7.
