# Supplementary material for: A new AMPK isoform mediates glucose-restriction induced longevity non-cell autonomously by promoting membrane fluidity
Source: Nat Commun. 2023 Jan 18;14:288. doi: 10.1038/s41467-023-35952-z (PMC9849402; doi:10.1038/s41467-023-35952-z)
Supplement: Supplementary file 3 — Description of Additional Supplementary Files [file 41467_2023_35952_MOESM3_ESM.pdf]

Title: Supplementary Movie. 1

Description: Fluorescence Recovery after Photobleaching measurements of worms expressing *glo-1p*::GFP::*ras-2* CAAX on AL diets (Related to Fig. 7a).

Fluorescence intensity was measured for 90 s every 2 s. The movie was filmed with 5.00 fps frame rate using a Zeiss LSM800 confocal microscope and Zen software (Zeiss).

Title: Supplementary Movie. 2

Description: Fluorescence Recovery after Photobleaching measurements of worms expressing *glo-1p*::GFP::*ras-2* CAAX on GR diets (Related to Fig. 7a).

Fluorescence intensity was measured for 90 s every 2 s. The movie was filmed with 5.00 fps frame rate using a Zeiss LSM800 confocal microscope and Zen software (Zeiss).

Title: Supplementary Movie. 3

Description: Fluorescence Recovery after Photobleaching measurements of *aak-2* mutant worms expresssing *glo-1p*::GFP::*ras-2* CAAX on AL diets (Related to Fig. 7d).

Fluorescence intensity was measured for 90 s every 2 s. The movie was filmed with 5.00 fps frame rate using a Zeiss LSM800 confocal microscope and Zen software (Zeiss).

Title: Supplementary Movie. 4

Description: Fluorescence Recovery after Photobleaching measurements of *aak-2* mutant worms expresssing *glo-1p*::GFP::*ras-2* CAAX on GR diets (Related to Fig. 7d).

Fluorescence intensity was measured for 90 s every 2 s. The movie was filmed with 5.00 fps frame rate using a Zeiss LSM800 confocal microscope and Zen software (Zeiss).

Title: Supplementary Movie. 5

Description: Fluorescence Recovery after Photobleaching measurements of *aak-2;aak-2a::gfp* mutant worms expresssing *glo-1p*::GFP::*ras-2* CAAX on AL diets (Related to Fig. 7e).

Fluorescence intensity was measured for 90 s every 2 s. The movie was filmed with5.00 fps frame rate using a Zeiss LSM800 confocal microscope and Zen software (Zeiss).

Title: Supplementary Movie. 6

Description: Fluorescence Recovery after Photobleaching measurements of *aak-2;aak-2a::gfp* mutant worms expresssing *glo-1p*::GFP::*ras-2* CAAX on GR diets (Related to Fig. 7e).

Fluorescence intensity was measured for 90 s every 2 s. The movie was filmed with5.00 fps frame rate using a Zeiss LSM800 confocal microscope and Zen software (Zeiss).
