# Supplementary material for: A new AMPK isoform mediates glucose-restriction induced longevity non-cell autonomously by promoting membrane fluidity
Source: Nat Commun. 2023 Jan 18;14:288. doi: 10.1038/s41467-023-35952-z (PMC9849402; doi:10.1038/s41467-023-35952-z)
Supplement: Supplementary file 10 — Reporting Summary [file 41467_2023_35952_MOESM10_ESM.pdf]

nature portfolio

Corresponding author(s): Geum-Sook Hwang, Eun-Soo Kwon

Last updated by author(s): Dec 13, 2022

Reporting Summary

Nature Portfolio wishes to improve the reproducibility of the work that we publish. This form provides structure for consistency and transparency in reporting. For further information on Nature Portfolio policies, see our Editorial Policies and the Editorial Policy Checklist.

Statistics

For all statistical analyses, confirm that the following items are present in the figure legend, table legend, main text, or Methods section.

n/a	Confirmed	
☐	☒	The exact sample size (n) for each experimental group/condition, given as a discrete number and unit of measurement
☐	☒	A statement on whether measurements were taken from distinct samples or whether the same sample was measured repeatedly
☐	☒	The statistical test(s) used AND whether they are one- or two-sided<br>*Only common tests should be described solely by name; describe more complex techniques in the Methods section.*
☐	☒	A description of all covariates tested
☒	☐	A description of any assumptions or corrections, such as tests of normality and adjustment for multiple comparisons
☐	☒	A full description of the statistical parameters including central tendency (e.g. means) or other basic estimates (e.g. regression coefficient) AND variation (e.g. standard deviation) or associated estimates of uncertainty (e.g. confidence intervals)
☐	☒	For null hypothesis testing, the test statistic (e.g. F, t, r) with confidence intervals, effect sizes, degrees of freedom and P value noted<br>*Give P values as exact values whenever suitable.*
☒	☐	For Bayesian analysis, information on the choice of priors and Markov chain Monte Carlo settings
☒	☐	For hierarchical and complex designs, identification of the appropriate level for tests and full reporting of outcomes
☒	☐	Estimates of effect sizes (e.g. Cohen's d, Pearson's r), indicating how they were calculated

Our web collection on statistics for biologists contains articles on many of the points above.

Software and code

Policy information about availability of computer code

Data collection	No software was used for data collection
Data analysis	lifespan assays<br>OASIS(https://sbi.postech.ac.kr/oasis/) was used for a log-rank test.<br><br>Fat store was analyzed with ImageJ (1.53e version)

For manuscripts utilizing custom algorithms or software that are central to the research but not yet described in published literature, software must be made available to editors and reviewers. We strongly encourage code deposition in a community repository (e.g. GitHub). See the Nature Portfolio guidelines for submitting code & software for further information.

Data

Policy information about availability of data

All manuscripts must include a data availability statement. This statement should provide the following information, where applicable:

- Accession codes, unique identifiers, or web links for publicly available datasets
- A description of any restrictions on data availability
- For clinical datasets or third party data, please ensure that the statement adheres to our policy

Source data including the individual P values, whole western blot image and lifespan raw data are provided with this paper.

Human research participants

Policy information about studies involving human research participants and Sex and Gender in Research.

Reporting on sex and gender	N/A
Population characteristics	N/A
Recruitment	N/A
Ethics oversight	N/A

Note that full information on the approval of the study protocol must also be provided in the manuscript.

Field-specific reporting

Please select the one below that is the best fit for your research. If you are not sure, read the appropriate sections before making your selection.

[x] Life sciences [ ] Behavioural & social sciences [ ] Ecological, evolutionary & environmental sciences

For a reference copy of the document with all sections, see nature.com/documents/nr-reporting-summary-flat.pdf

Life sciences study design

All studies must disclose on these points even when the disclosure is negative.

Sample size	Sample size was determined based on similar experiments from previous publications. No statistical method was used to predetermine sample size. In general all the experiments were performed with at least two independent biological trials.
Data exclusions	No data were excluded from the study
Replication	The data in this study were reproducible and repeated with at least two independent reproducible replicates.
Randomization	The samples were randomly allocated into experimental groups.
Blinding	Lifespan assays, metabolite analysis, paralysis assays, heat resistance assays, brood size assays, lipidomic, fat staining and FRAP were performed double-blindly and by independent researchers.<br>Western blotting was not performed blindly due to technical issue (order of loading samples).<br>Lifespan assays on distinct diets were not performed blindly due to apparent differences between control diets and glucose-restricted diets.

Reporting for specific materials, systems and methods

We require information from authors about some types of materials, experimental systems and methods used in many studies. Here, indicate whether each material, system or method listed is relevant to your study. If you are not sure if a list item applies to your research, read the appropriate section before selecting a response.

Materials & experimental systems

n/a	Involved in the study	
[ ]	[x]	Antibodies
[x]	[ ]	Eukaryotic cell lines
[x]	[ ]	Palaeontology and archaeology
[ ]	[x]	Animals and other organisms
[x]	[ ]	Clinical data
[x]	[ ]	Dual use research of concern

Methods

n/a	Involved in the study	
[x]	[ ]	ChIP-seq
[x]	[ ]	Flow cytometry
[x]	[ ]	MRI-based neuroimaging

Antibodies

Antibodies used	Primary antibodies: GFP (ab290, Abcam), β-actin (ab133626, Abcam)<br>Secondary antibodies: Goat anti-rabbit IgG HRP conjugated (#31460, ThermoFisher)
Validation	All antibodies used in this study were validated by the manufacturer company. Validation data / citation can be found on the

manufacturer website by searching the catalog number provided in materials and methods section.
GFP (ab290, Abcam): https://www.abcam.com/gfp-antibody-ab290.html,
β-actin (ab133626, Abcam): https://www.citeab.com/antibodies/713976-ab133626-anti-beta-actin-antibody-epr6255,
Goat anti-rabbit IgG HRP conjugated (#31460, ThermoFisher): https://www.thermofisher.com/antibody/product/Goat-anti-Rabbit-IgG-H-L-Secondary-Antibody-Polyclonal/31460

Animals and other research organisms

Policy information about studies involving animals; ARRIVE guidelines recommended for reporting animal research, and Sex and Gender in Research

Laboratory animals	Larval and adult Caenorhabditis elegans were used in this study. These include wild-type N2(Bristol), RB755, RB2240, CL2006, DA1116, GR1307, VC222, KQ1366, ZG31, RB754, VC199, PS3551, EU31, KU4, VC1024, RB2547, MQ887, NL2099, TU3401, VP303, MT7929, EG9631, CB450, CB1091, DA509, KP2018, RB993, MT14984, CB1112, WBM55, CU5991, CU6372, WBM409, XA7702, BX107, BX106, BX153, BX160, BX110, BX156, QC114, QC129. The double or triple mutants were generated in classical genetic methods, followed by geno-typing. Transgenic animals such as AAK-2::GFP strains were generated by micro-injection. day 5 adult worms were used for heat resistance assays. For GFP images and fat staining, day1 adult animals were used. For AAK-2 isoform western blotting, larvae enriched populations were used. For FRAP assay and C-Laurdan staining, L2 staged worms were used.
Wild animals	No wild animals was used in this study
Reporting on sex	hermaphrodites and males of Caenorhabditis elegans were used for lifespan assays. Male C. elegans were distinguished by mating structures such as the blunt tail with fan, rays and hook.
Field-collected samples	No field-collected samples was used in this study
Ethics oversight	No ethics approval was required for C. elegans

Note that full information on the approval of the study protocol must also be provided in the manuscript.
